# Preclinical and Clinical Evaluation of Magnetic-Activated Cell Separation Technology for CTC Isolation in Breast Cancer

**DOI:** 10.3389/fonc.2020.554554

**Published:** 2020-09-15

**Authors:** Taja Lozar, Tanja Jesenko, Veronika Kloboves Prevodnik, Maja Cemazar, Violeta Hosta, Anja Jericevic, Natasa Nolde, Cvetka Grasic Kuhar

**Affiliations:** ^1^Faculty of Medicine, University of Ljubljana, Ljubljana, Slovenia; ^2^Department of Experimental Oncology, Institute of Oncology Ljubljana, Ljubljana, Slovenia; ^3^Department of Cytopathology, Institute of Oncology Ljubljana, Ljubljana, Slovenia; ^4^Faculty of Health Sciences, University of Primorska, Izola, Slovenia; ^5^Department of Dermatovenereology, University Medical Centre Ljubljana, Ljubljana, Slovenia; ^6^Department of Medical Oncology, Institute of Oncology Ljubljana, Ljubljana, Slovenia

**Keywords:** liquid biopsy, magnetic-activated cell separation, circulating tumor cells, morphology, breast cancer

## Abstract

Circulating tumor cell (CTC) count is an independent prognostic factor in early breast cancer. CTCs can be found in the blood of 20% of patients prior to neoadjuvant therapy. We aimed to assess the suitability of magnetic-activated cell separation (MACS) technology for isolation and cytological characterization of CTCs. In the preclinical part of the study, cell lines were spiked into buffy coat samples derived from healthy donors, and isolated using MACS. Breast cancer cells with preserved cell morphology were successfully isolated. In the clinical part, blood for CTC isolation was drawn from 44 patients with early and locally advanced breast cancer prior to neoadjuvant chemotherapy. Standard Giemsa, Papanicolaou and pancytokeratin staining was applied. 2.3% of samples contained cells that meet both the morphological and immunocytochemical criteria for CTC. In 32.6% of samples, partially degenerated pancytokeratin negative cells with morphological features of tumor cells were observed. In 65.1% of samples, CTCs were not found. In conclusion, our results demonstrate that morphologically intact tumor cells can be isolated using MACS technology. However, morphologically intact tumor cells were not detected in the clinical part of the study. At present, MACS technology does not appear suitable for use in a clinical cytopathology laboratory.

## Introduction

Circulating tumor cells (CTCs) are believed to be an intermediate step in the metastatic cascade. During growth of the primary or metastatic tumor, epithelial tumor cells may leave the tumor site, invade into a blood or lymphatic vessel and circulate in the bloodstream to disseminate throughout the body, potentially becoming the source of cancer metastases (1, 2). These cells are known to exhibit distinct antigenic and morphological characteristics, such as high levels of epithelial-mesenchymal plasticity, larger size compared to white blood cells (WBC), larger nuclear/cytoplasmic (N/C) ratio, and distinct nuclear morphology (3–6). Multiple studies have demonstrated CTC count is an independent prognostic factor in various types of cancer, including breast cancer (7–12). In metastatic breast cancer, the presence of 5 or more CTCs per 7.5 ml of blood has been associated with a reduction in progression free survival and overall survival, as well as with higher disease progression and mortality (13–21). In patients with early breast cancer, CTCs can be found in the blood of roughly 20% of patients prior to neoadjuvant therapy (22–26). The presence of CTCs in peripheral blood has been linked to lymph node metastasis and demonstrated significant prognostic impact on disease-free and overall survival (27). In addition, CTC detection shows promise for assessing treatment efficacy in neoadjuvant therapy (28). CTC detection before neoadjuvant setting carries an independent negative prognostic value for a reduced disease-free and overall survival (29), while not being associated with pathologic complete response (25, 30). Taken together, this data indicates that breast cancer patients before neoadjuvant setting may represent a population that might benefit most from CTC diagnostics.

Reliable isolation and detection of CTCs remains a diagnostic challenge. The concentration of CTCs from epithelial-derived cancers has been found to be extremely low (1 to 10 cells per ml blood), presumably due to their short half-life in the blood stream and proneness to constant genotypic and phenotypic changes (4–6, 31–33). In recent years, several methods for CTC isolation and detection have been established and these have been described elsewhere (34). To this day, the CellSearch^®^ is the only Food and Drug Administration approved assay to be used as a prognostic tool in the management of breast cancer, prostate cancer and colorectal cancer patients (11, 35–38). The CellSearch^®^ along with many other isolation technologies uses magnetic beads covered with anti-epithelial cell adhesion molecule (EpCAM) antibodies for positive selection of tumor cells of epithelial origin. CTC separation and magnetic bead washing is followed by detection based on immunofluorescent cytokeratin (CK), 4,6-diamidino-2-phenylindole (DAPI) and CD45 (common lymphocyte antigen) staining and subject to single cell level semi-automated morphologic analysis. CTCs can be identified as CK and DAPI positive, CD45 negative cells with a diameter larger than 4 μm (38). While this strategy has proven to have prognostic value (11, 36, 38, 39), epithelial cell marker dependent criteria have been associated with some degree of underestimation of CTC numbers (40) related to the epithelial-mesenchymal transition phenotype (41).

As previously pointed out by Marrinucci et al. (42), a limitation of fluorescence-based methods is that morphological features of isolated CTCs are difficult to evaluate and compare to the results obtained by routine methods used in cytopathology. Moreover, immunocytochemistry (ICC) cannot be used either. ICC is one of the most widely used tools in cancer diagnostics, mostly because of its role in immunophenotypic characterization of tumor cells before more costly molecular methods. The cytopathology laboratory has been proposed as the ideal environment when attempting to implement CTC enumeration into clinical practice. Besides existing infrastructure, the cytopathologists also possess the required expertise for CTC evaluation, such as integrating morphologic, immunophenotypic and molecular data to provide the patient and the referring clinician with a final diagnosis (43).

In this study, a CTC isolation protocol was designed and tested in a pre-clinical and clinical setting. Positive-selection based magnetic-activated cell sorting (MACS) technology using EpCAM conjugated magnetic beads in the magnetic field was used for CTC isolation (72). The technology was previously reported successful in breast cancer CTC enrichment (44). The aim of the study was to assess the suitability of the technology for CTC diagnostics in the routine cytopathology laboratory. The pre-clinical part of the study aimed to determine the method’s sensitivity and specificity, and evaluate if the isolation protocol preserves cancer cell morphology. The aim of the clinical part was CTC isolation and subsequent morphological analysis in patients with early breast cancer prior to neoadjuvant chemotherapy.

## Materials and Methods

### Study Design

The study was designed as a preclinical evaluation followed by a prospective clinical trial, and was conducted at the Institute of Oncology Ljubljana, Slovenia. As part of the preclinical evaluation, buffy coat (BC) from healthy donors was spiked with cultured breast cancer cells. The isolation of cancer cells, Giemsa, Papanicolaou and ICC staining, and subsequent microscopical evaluation with recovery rate calculation, were performed at the Department of Cytopathology. To determine the specificity of the isolation protocol, lung fibroblasts were also spiked into BC, which was followed by isolation and an evaluation of the enriched fraction.

In the clinical part of the study, breast cancer patients diagnosed with clinically lymph node-positive disease and/or tumor with a diameter larger than 2 cm were included in the study. Other inclusion criteria were: age 18 years or older, cytologically confirmed breast cancer, scheduled to receive neoadjuvant chemotherapy, and patient has not yet had core needle biopsy. Data on patient and tumor characteristics and pathological response following neoadjuvant chemotherapy were collected from patient charts. Pathological response was evaluated in tumor and lymph nodes after neoadjuvant chemotherapy. Pathological complete response (pCR) in the tumor was defined as absence of invasive carcinoma in the breast after neoadjuvant chemotherapy. pCR in lymph nodes was defined as absence of invasive carcinoma in the lymph nodes after neoadjuvant chemotherapy. The patients’ complete blood counts were examined for the presence of immature erythro-myeloid progenitor cells.

### Ethics Statement

The study was reviewed and approved by the National Medical Ethics Committee at the Slovenian Ministry of Health (ref. nr. 0120-133/2017-2 and 0120-150-2019/4) and was conducted in accordance with the Declaration of Helsinki. All enrolled patients signed an informed consent form.

### Blood Samples

In the preclinical part of the study, BC from the blood of anonymous healthy donors was obtained from the Blood Transfusion Centre of Slovenia. BC samples were then diluted using a separation buffer [MACS BSA Stock Solution and autoMACS Rinsing Solution, 1:20, Ph 7.2 phosphate buffer solution (PBS), 0.5% bovine serum albumin (BSA), 2 mM ethylenediaminetetraacetic acid (EDTA)]. Reference values for WBC content in BC solutions were obtained from the literature (45) and a dilution coefficient required to achieve a leukocyte/ml concentration in line with reference values for whole blood samples (46) was calculated (1:5).

In the clinical part of the study, patients donated an additional whole peripheral blood sample (10 ml) during a regular blood draw prior to core-needle biopsy. The study sample for CTC isolation was collected last during routine laboratory blood testing to avoid any contamination with skin epithelial cells during needle insertion. The sample was stored in an EDTA-containing collection tube and processed within 1 h.

### Cell Culture and Processing

Human epithelial breast cancer cell line MCF7 (ATCC^®^ HTB-22^TM^, ATCC, Manassas, United States) and human mesenchymal fibroblast cell line Wi-38 (ATCC^®^ CCL75^TM^, ATCC) were purchased directly from ATCC. The cells were propagated in culture using the AMEM medium (Gibco, Thermo Fisher Scientific, Waltham, MA, United States) with 5% bovine serum, glutamine (10 Mm, Gibco), crystacillin (100 U/ml; Pliva d.d., Zagreb, Croatia) and gentamicin (50 μg/ml; Krka d.d., Novo mesto, Slovenia). The cells were cultured in a 5% CO_2_ humidified incubator at 37°C. The cells were grown as a monolayer until they reached at least 80% confluence. Afterward, the medium was removed, the cells were first washed with PBS and afterward detached from the surface with 0.25% trypsin/EDTA in Hank’s buffer (Gibco). After detachment, the cells were collected, counted, and a cell suspension with appropriate cell density was prepared. The cultured cells (100 or 1000 cells) were spiked into 10 ml of diluted BC and processed within 1 h identically to the patients’ whole blood specimens. Altogether, 14 BC samples were spiked with MCF7 cells and eight samples were spiked with Wi-38 cells.

### Isolation Protocol

A positive selection-based MACS technology was used (Miltenyi Biotec, Bergisch Gladbach, Germany) following the manufacturer’s instructions. The sample was first filtered through a 30 μm pre-separation filter (Miltenyi Biotec). The sample was then incubated with anti-EpCAM magnetic beads (StraightFrom^®^ Whole Blood CD326 (EpCAM) MicroBeads, Miltenyi Biotec, 50 μl/ml blood, 30 min, 2–8°C) for magnetic labeling, followed by centrifugation (445 rpm, 10 min) and resuspension of the cell fraction with a separation buffer. Magnetic separation was performed using a column containing a matrix composed of ferromagnetic spheres (Whole Blood Columns, Miltenyi Biotec) placed in a separator (QuadroMACS Separator, Miltenyi Biotec). This was followed by the elution step (Whole Blood Column Elution Buffer, Miltenyi Biotec, 5 ml), yielding an enriched, positively selected fraction. All collection tubes were extensively washed with separation buffer throughout the procedure in order to avoid any potential cell loss.

### Staining and Cytopathological Evaluation

Two slides for microscopical examination (cytospins) were prepared from each isolated fraction by a cytocentrifuge (Thermo Scientific Shandon Cytospin^®^ 4 Cytocentrifuge, Waltham, MA, United States) at 1000 rpm for 4 min at room temperature. One cytospin was air dried at room temperature for a minimum of 30 min and stained with Lopez Cordosa Giemsa (47). The second cytospin was fixed (Delunay, 30 min) and stained with Papanicolaou (PAP) using an automated stainer Leica Multistainer ST5020 (Leica Microsystems, Buffalo Grove, IL, United States). The stained cytospins were then screened by a cytopathologist. Morphological features, such as cell size, amount and quality of cytoplasm, cytoplasmic inclusions, the size of the nucleus and nucleolus, nuclear/cytoplasmic (N/C) ratio, quality of the chromatin and nuclear membrane irregularities were examined. If cells with morphological features in favor of CTC were observed, the PAP stained cytospin was ICC stained with anti CK monoclonal antibody (CK AE1/AE3, ref. nr. M3515, dilution 1:500, Dako, Agilent, Santa Clara, CA, United States). If morphological analysis was inconclusive, additional staining for CD45 (LCA, clone 2B11 + PD7/26, ref. nr. M0701, dilution 1:1000, Dako) was performed on previously CK stained slide to exclude the hematopoietic origin of the cells in question. In slides derived from BC samples spiked with fibroblasts, vimentin expression was ICC determined (clone V9, ref. nr. M0725, dilution 1:4000, Dako). All ICC staining was performed on BenchMark ULTRA imunohistochemical stainer (Ventana, Roche Diagnostics, Oro Valley, AZ, United States) following the manufacturer’s instructions.

Cytospins that contained cells with morphological features in favor of CTC were documented and sent for an independent review to three additional cytopathologists. Based on their consensus, the slides were labeled as: (1) CTC positive (canonical), if malignant morphological features as well as CK positivity were observed in at least one cell, (2) Non-canonical CTCs, if malignant morphological features were observed in at least one cell, but there was no CK expression, and (3) CTC negative, if there were no malignant morphological features suggestive of tumor cells and no CK expression.

### Statistical Analysis

The patient’s categorical characteristics were presented as frequencies and proportions. Age was presented as median and range. Pearson’s chi-square test was used for statistical comparisons; a *p*-value of ≤0.05 was considered statistically significant. All statistical analysis was performed using SPSS v.24.0 (IBM Corp., Armonk, NY, United States).

## Results

### Isolation Sensitivity and Specificity

The results of sensitivity and specificity analysis are presented in Table 1. To evaluate the method’s sensitivity, 14 BC samples were spiked with Michigan Cancer Foundation 7 (MCF7) cells and examined before and after the MACS isolation procedure. Seven BC samples were spiked with 1000 MCF7 cells, and 7 samples with 100 MCF7 cells. The recovery rate for 1000 spiked MCF7 cells was 34.31 (95% CI 33.20–35.44) and 28.14 (95% CI 24.84–31.63) for 100 spiked MCF7 cells. The sensitivity of our isolation protocol was 34% (95% CI 32.7–34.82%).

**TABLE 1:** Sensitivity and specificity analysis.

Spiked cells (N)	Experiments (N)	Recovery rate (%)	95% confidence interval
MCF7			
1000	7	34.3	33.2–35.4
100	7	28.1	24.8–31.6
Wi-38			
1000	4	0	–
100	4	0	–

*Spiked Michigan Cancer Foundation 7 (MCF7) cells were used to determine the sensitivity of the method. Spiked fibroblasts (Wi-38) were used to determine the specificity of the method.*

**FIGURE 1:**
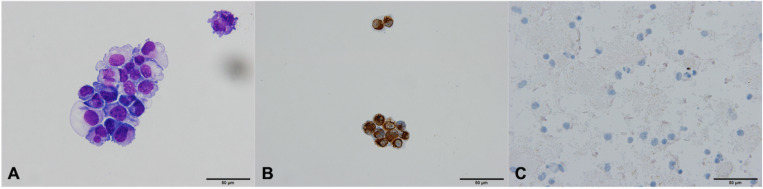
Fibroblasts Wi-38 before and after isolation using MACS technology, ×60. **(A)** Giemsa stain, before isolation. **(B)** Positive vimentin stain, before isolation. **(C)** Negative vimentin staining after isolation, demonstrating absence of Wi-38 fibroblasts.

To evaluate the specificity of the method, 8 BC samples were spiked with Wi-38 fibroblasts and examined before (Figures 1A,B) and after the MACS isolation procedure (Figure 1C). Four samples were spiked with 100 cells, and four samples were spiked with 1000 cells. No fibroblasts were observed in samples undergoing MACS isolation (Figure 1C). The calculated specificity was 100% (95% CI 99.92 − 100.00%) (Table 1), since no fibroblasts were observed in samples undergoing MACS isolation (Figure 1C).

### Preservation of Morphology of Cultured Tumor Cells

**FIGURE 2:**
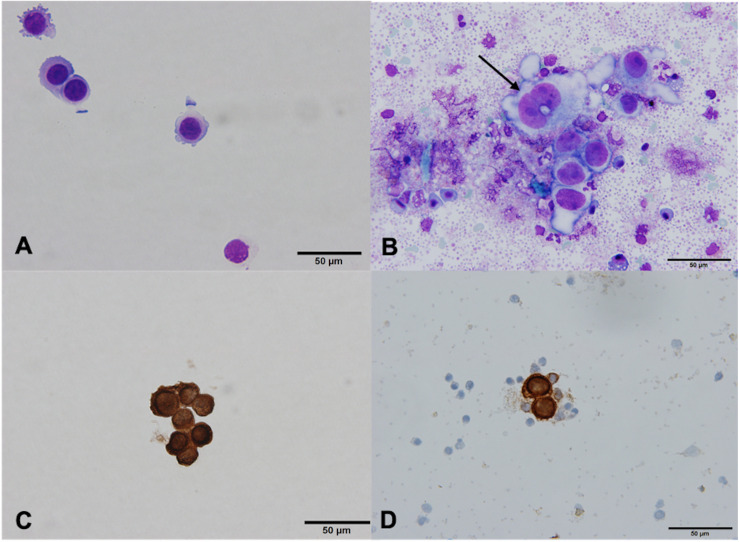
MCF7 cells before and after isolation using MACS technology, ×60. **(A)** Giemsa stain, before isolation. **(B)** Giemsa stain, after isolation. MCF7 cells vary in size and shape, and have prominent nucleoli (arrow). Bare nuclei and partially degenerated/apoptotic white blood cells (WBC) and red blood cells (RBC) are in the background. **(C)** Positive cytokeratin (CK) stain, before isolation. **(D)** Positive CK stain, after isolation. MCF7 cells are clearly CK positive, surrounded by CK negative WBC and RBC.

The isolated MCF7 cells in 14 BC samples were morphologically well preserved and resembled MCF7 cells that had not undergone MACS isolation procedure (Figures 2A,B). The isolated cells were very large compared to WBC, with high N/C ratios and scant to moderate amounts of pale basophilic, vacuolated cytoplasm with cell membrane irregularities and cytoplasmic blebs (Figure 2B). The nuclei were oval, hyperchromatic and pleomorphic. In some cells, nucleoli were visible (Figure 2B). The cells had intact cell membrane and were not damaged or apoptotic. Based on the morphology of isolated MCF-7 cells, we believe the cells are viable after isolation using the MACS technology. Substantial amounts of white blood cells (WBC) and platelets were observed in the background after isolation (Figure 2B). In MCF7 cells, CK staining was positive in all examined samples (Figures 2C,D).

### Prevalence and Morphology of CTCs in Early Breast Cancer

The characteristics of 43 out of the 44 patients included in the clinical part of the study are summarized in Table 2. The sample of one patient had to be excluded from the analysis since more than 1 h had passed between the blood draw and isolation.

**TABLE 2:** Patient characteristics.

Characteristic*	Number of patients *N* = 43
Age (years)	46 (26, 74)
Tumor histology	
IDC	37 (86)
ILC	5 (11.7)
Mucinous carcinoma	1 (2.3)
Tumor grade	
II	13 (30.2)
III	30 (69.8)
Estrogen receptor status	
Negative	14 (32.6)
Positive	29 (67.4)
Progesterone receptor status	
Negative	21 (48.8)
Positive	14 (32.6)
HER2 receptor status	
Negative	29 (67.4)
Positive	14 (32.6)
MIB-1 expression	
Negative (≤10%)	5 (11.6)
Positive (>10%)	38 (88.4)
Molecular subtype	
Triple negative	8 (18.5)
HER2 positive	8 (18.5)
HER2 positive Luminal	6 (14)
Luminal B-like	18 (42)
Luminal-A-like	3 (7)
Tumor stage	
T1	6 (14)
T2	23 (53.4)
T3	10 (23.3)
T4	4 (9.3)
Nodal involvement	
No	7 (16.3)
Yes	36 (83.7)
Breast cancer stage	
II	29 (67.4)
III	12 (27.9)
IV	2 (4.7)
Initial treatment	
Neoadjuvant chemotherapy	34 (79.1)
Surgery	9 (20.9)
Presence of ≥1 CTC	
Negative	28 (65.1)
Canonical	1 (2.7)
Non-canonical	14 (32.2)
pCR after neoadjuvant chemotherapy	
Tumor	13 (30.2)
Lymph nodes	17 (39.5)
Lymph nodes negative before and after	5 (11.6)
neoadjuvant chemotherapy	

*Presented as number (percentage), except for age, which is presented as median (range). N, number of patients; IDC, invasive ductal carcinoma; IDL, invasive lobular carcinoma; CTC, circulating tumor cells; HER2, human epidermal growth factor receptor 2; pCR, pathological complete response.

**FIGURE 3:**
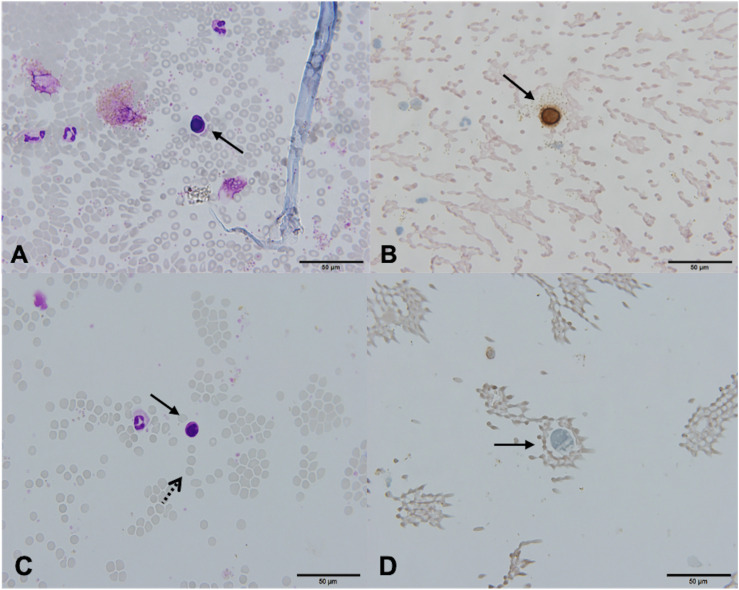
Canonical [Patient 43, **(A,B)**] and non-canonical [Patient 39, **(C,D)**] circulating tumor cells (CTCs), ×60. **(A)** A morphologically preserved cell with features suggestive for CTC, Giemsa stain. **(B)** Positive immunocytochemical staining for cytokeratin (CK) on a Papanicolaou stained cytospin. **(C)** A cell resembling a lymphoid cell (arrow). Its diameter is three times the size of the surrounding erythrocytes (dotted arrow). The nucleus is ovoid with altered chromatin structure and high N/C ratio. Giemsa stain. **(D)** Cells exhibiting negative staining for CK and CD45 (arrow).

In one patient (2.3%), the cytospin contained cells that meet the morphological and immunocytochemical criteria for CTC identification (CTC positive, Figures 3A,B). These canonical CTCs were larger than the surrounding WBC, roughly 3–4 times the size of a mature lymphocyte. The cells were oval in shape with scant eosinophilic cytoplasm, a high N/C ratio, and rounded nuclei with dispersed chromatin. The nucleoli were not visible (Figure 3A). ICC staining for CK was positive (Figure 3B).

**FIGURE 4:**
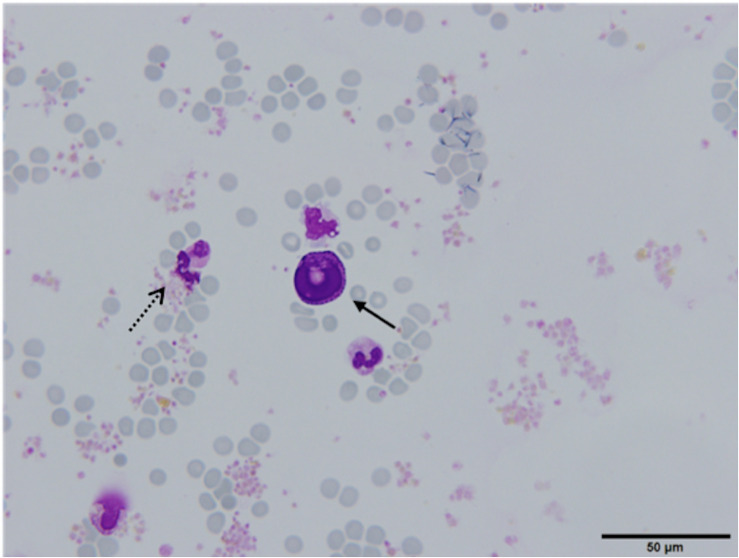
(Partially) degenerated neutrophils (dotted arrow) with a non-canonical circulating tumor cell (CTC) in the middle (arrow), ×60. The non-canonical CTC was notably larger in size compared to mature WBC (diameter 22.53 μm) and exhibits features suggestive of degeneration (arrow). The cytoplasm was scant, eosinophilic, with an oval nucleus with degenerative intranuclear vacuoles and ill-defined chromatin structure, Giemsa stain.

**FIGURE 5:**
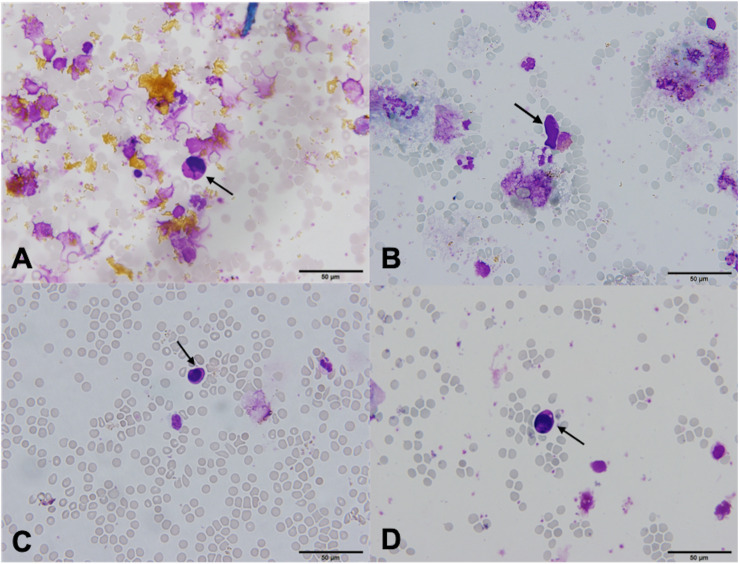
**(A–D)** Non-canonical circulating tumor cells (CTCs), ×60. Cells with scant eosinophilic cytoplasm, an increased nuclear/cytoplasmic ratio and degenerated nuclei with loss of chromatin structure, Giemsa stain [**(A,C,D)**, arrows]. Single bare nuclei were also observed [**(B)**, arrow]. Yellow granular material was observed in the background of some samples **(A)**.

In 28 (65.1%) patients, no CTCs were detected. However, in 14 (32.6%) patients, the samples contained cells that were partially degenerated, CK and CD45 negative, but showed morphological features of tumor cells (non-canonical CTCs, Figures 3C,D, 4). We failed to find immature WBC in the complete and differential blood counts of these patients, which could resemble non-canonical CTCs. Furthermore, these non-canonical CTCs did not resemble the surrounding mature WBC. Their morphological features were: scant eosinophilic cytoplasm with vacuoles and eosinophilic inclusions, large nuclei, high N/C ratio, and irregular nuclear contours with loss of chromatin structure (Figures 3C,D). WBC, erythrocytes and thrombocytes were observed in the background of all slides after CTC isolation (Figures 4, 5). Bare oval nuclei were also observed (Figure 5B).

### Correlation With Tumor Characteristics and Pathological Response

Circulating tumor cells (canonical and non-canonical) were more often detected in patients undergoing neoadjuvant treatment than surgery (41% vs. 11%; *p* = 0.092). CTCs were detected more often in HER2 positive patients than in HER2 negative patients (50% vs. 28%), but this correlation was not statistically significant (Pearson’s Hi-square *p* = 0.148). There was no correlation between age, histology, grade, hormone receptor status, tumor stage, nodal involvement and the presence of CTCs. In 34 patients treated with neoadjuvant chemotherapy, pCR in the breast was achieved in 35% of CTC positive and in and 40% of CTC negative samples. In addition, pCR in the lymph nodes was observed in 50% of CTC positive and CTC negative samples. The presence of CTCs after neoadjuvant chemotherapy was not evaluated.

## Discussion

This study aimed to evaluate the feasibility of the MACS technology for CTC isolation and subsequent cytopathological examination in the routine cytopathological laboratory setting in early breast cancer. The present study is one of the few published studies on the morphology of breast cancer CTCs. It is also one of the few published studies using standard cytopathological techniques for CTC preparation and morphological analysis using light microscopy. The results of this study show that MACS technology preserves the morphology of breast cancer cells from MCF7 cell line, however, this was not observed in CTCs from breast cancer patients. Based on the findings of this study, we believe isolation with MACS technology followed by preparation of standard cytological slides is at present not yet suitable for routine CTC diagnostics in early breast cancer patients.

In clinical trials looking at the performance of CTC isolation methods by spiking cultured tumor cells to whole blood or peripheral blood mononuclear cell suspensions, the preservation of morphology was usually examined using fluorescent microscopy, assessing basic features, such as cell size and N/C ratio (3). In the present study, standard light microscopy was used for such examination. The MCF7 cell line was chosen as it is canonical for breast cancer and the most commonly used breast cancer cell line in the literature (48, 49), and because our cytopathological laboratory has vast experience with its preparation and light microscopy examination.

The sensitivity of our method as investigated in the preclinical part of the study was found to be lower as previously reported. The recovery rates for positive selection-based isolation methods obtained by spiking cultured breast cancer cells into whole peripheral blood range from 60 to 100% (50–52). One of the first studies evaluating the performance of immunomagnetic separation using breast cancer cell lines and spiking 1000, 100, and 10 cells found a 75% recovery rate, which is higher than the recovery rate reported in the present study (34%) (53). Although extensive washing to prevent potential cell loss was applied, the non-automated handling of samples in our protocol may have resulted in significant cell loss. Furthermore, the manufacturer’s protocol is optimized for whole blood samples, therefore the lower sensitivity could also be attributed to the use of diluted BC samples in preclinical part of this study. Unfortunately, we did not plan to obtain whole blood samples from healthy volunteers.

The main challenge we faced in the course of this study was the identification of cells that exhibited morphological features of malignancy while staining negative for CK. The criteria that were used to label the study samples were based on the presence of atypical morphology and CK positivity, similar to the criteria used by Tsutsuyama et al. (54). We identified one (2.3%) sample that could be labeled positive based on both criteria, and 65.1% of samples that were labeled negative. Cells meeting only the morphological criteria were found in 32.6% of samples. We hypothesized about the origins of these cells. Firstly, they could belong to the erythro-myeloid progenitor cells in the blood of these patients. To rule out this possibility, complete and differential blood counts from a blood sample obtained on the same day as the study sample were examined. No immature erythro-myeloid progenitor cells were identified in any of the samples. Consequently, we hypothesize the observed cells exhibiting malignant features in study samples were in fact degenerated epithelial tumor cells. Our preclinical experiments also showed high isolation specificity, since no spiked lung fibroblasts were observed in the BC samples that underwent the isolation procedure. Based on these observations, the cells in question were labeled non-canonical CTCs.

Although the non-canonical CTCs exhibited morphological features of malignancy, they were severely degenerated (bare, oval nuclei, degenerative intracytoplasmic and intranuclear vacuoles, and nuclear fragmentation) and did not stain for CK. The observed poor preservation of CTCs in patients’ samples could be due to several possible reasons. CTCs could be damaged by shear stress while still circulating in the blood stream, or they could be circulating apoptotic cells. Additionally, CTCs appear to be more sensitive and fragile compared to cultured cells and, therefore, could be damaged more during the isolation procedure. Moreover, the morphology of isolated tumor cells further changed during cytospin preparation due to the centrifuge force used to transfer the cells to the slides. Such observations have also been reported in a Japanese study using a filtration device and the centrifugation method for cell transfer (54). The authors similarly assumed the centrifugation may have influenced cell morphology of isolated cells in their study.

The morphological characteristics of our CTCs, labeled as non-canonical CTCs, are in line with reported characteristics of CTCs in metastatic breast cancer, lung adenocarcinoma, prostate and colon cancer (42, 55–57). Similar to our study, CTCs with nuclear and cytoplasmic fragmentation and irregularities were observed in a study on the morphology of colon cancer CTCs (56). In CTCs from patients with lung adenocarcinoma, nuclear membrane irregularities and chromatin variation ranging from dense to dispersed chromatin with prominent nucleoli were also observed (55). A study by Marinucci et al. investigating the morphologic variation of CTCs in widely metastatic breast cancer is to the best of our knowledge the only study investigating the morphology of breast cancer CTCs. In this study, an immunofluorescent protocol targeting CK was used for identification and subsequent Wright-Giemsa staining and light microscopy analysis was performed on CK positive subset (42). In their cohort of CK positive cells, morphological analysis of Giemsa stained slides showed high degree of pleomorphism and several distinct CTC populations. Most notably, a large proportion (23%) of the isolated cells were larger than the surrounding WBCs, had high N/C ratio, a scant rim of amphophilic to eosinophilic cytoplasm and oval to lobulated nuclear contours. CTCs exhibiting early and late apoptotic changes making up over a half of the whole CTC population were also observed. These features are indeed in line with morphological findings observed in our study. Early apoptotic changes, such as condensation and shrinkage of nuclear material with loss of nuclear detail and formation of cytoplasmic inclusions, as well as late apoptotic changes, such as nuclear fragmentation, were observed in the entire population of cells that were labeled as non-canonical CTCs in our study. We hypothesize the above described apoptotic changes could be correlated to the loss of antigen characteristics and therefore the binding sites for ICC markers, however, all of the abovementioned studies observed such morphological changes in CK positive CTCs, indicating these changes did not affect the ICC staining process (42, 55, 56).

By performing complementary morphological evaluation in the process of CTC identification, false positive staining of hematopoietic cells due to unspecific binding of antibodies can be avoided (58). However, the utility of such evaluation remains limited because of various isolation methodologies, and their effects on the morphology of isolated cells (42). Another limitation of this approach is the fact that, in contrast to detailed morphological descriptions of primary or secondary tumors in histology and cytopathology, there is scant literature on the morphological features of CTCs and also no widely established morphologic criteria for the identification of CTCs during their travel through the bloodstream (55). As a result, most cytopathologists rely on their knowledge of tumor cell morphology observed in different cytological samples, such as fine needle aspirates, pleural effusions, ascites, bone marrow, etc. They may also rely on criteria used for diagnosing minimal residual disease. A list of objective criteria for the evaluation of minimal residual disease in bone marrow based on morphological analysis and ICC staining was published over a decade ago (59). A few years later, a classification by Fehm et al., integrating morphology, immunophenotyping and genotyping using fluorescent *in situ* hybridization was introduced (3). Criteria for morphological evaluation used in the present study, such as cell size larger than that of WBC, high N/C ratio, and irregular nuclear contours, were adopted from this classification (3). Based on these morphological criteria, only one patient sample contained cells that could unambiguously be classified as CTCs. An additional 35% of patient samples contained degenerated cells exhibiting morphological features of tumor cells, i.e., non-canonical CTC. However, based on the existing literature, the morphological criterion alone is not sufficient for definitive CTC identification. To conclude, the non-canonical CTC population observed in our study exhibits characteristics of malignant cells, however, this could not be confirmed with the available identification methods.

The main limitation of this study was the lack of an additional identification protocol that could confirm the cells labeled as non-canonical CTCs, such as genomic or transcriptomic analysis. In addition, flow cytometry could be considered for CTC isolation. Namely, fluorescence-activated cell sorting (FACS) enables simultaneous determination of multiple antigens, which allows sorting of CTCs for further analysis of different CTC populations. Similar to the MACS method, FACS also requires downstream morphological, immunophenotypical and molecular studies for CTC characterization.

Another limitation of the present study was that the viability of isolated cells was not evaluated. This could be performed using trypan blue staining. However, we believe this would not have been successful. Firstly, the number of isolated CTCs was too low. Secondly, we suspect that the morphological differentiation between suspected CTCs and WBCs would not be reliable using trypan blue staining. However, based on the morphology of MCF7 cells on samples stained with Giemsa and PAP, we believe the isolated cells retain viability after MACS isolation.

Furthermore, this study was performed on a patient cohort with early breast cancer. Although proven to be a prognostic factor, CTCs can only be found in about 20% of this patient population (10, 27, 60–62). In contrast, between 50 and 70% of metastatic breast cancer patients have detectable CTCs (38, 63). The low numbers of CTCs found in our study could be explained by the high proportion of patients with low stage tumors and low disease stage. Over a half of the patients included in this study had stage II disease, indicating low systemic disease burden. With this in mind, future evaluations of clinical suitability of CTC isolation method should be conducted in a patient cohort with more advanced disease stage, such as inflammatory or metastatic breast cancer, where higher CTC prevalence is expected.

Our study found no clinicopathological correlations with the presence of canonical or non-canonical CTCs before neoadjuvant setting, and no impact of detected CTCs on pCR. Published literature has shown correlations with tumor size (23, 64), tumor stage, and molecular classification (64). Until recently, no association between CTC detection prior to neoadjuvant therapy and pathological response in early breast cancer has been shown (23, 29). Recent evidence suggests conversion from CTC positive to CTC negative state after neoadjuvant therapy may be associated with some degree of pathological response (64). As this was a preliminary study in a small patient cohort, no extended protocol was in place to investigate prognostic relevance or monitor CTC dynamics longitudinally to make our data comparable to published literature. Investigating the method’s feasibility and CTC prevalence were our primary end points, and because of low numbers of CTCs and observed degeneration, we feel the MACS isolation method is not sensitive or robust enough to be used for longitudinal monitoring.

At the time of designing this preliminary study, we adapted a commercially available method using a widely established epithelial selection marker. However, by only using epithelial cell surface marker based positive-selection and identification, a potentially more aggressive CTC population that has lost its epithelial markers and gained mesenchymal, can be missed (65, 66). High levels of phenotypic heterogeneity among CTCs has been reported (57, 67), and while most current CTC detection methods only target EpCAM and/or CK to enrich epithelial CTCs, they may fail to recognize other CTC phenotypes that lack expression of these markers. Since the present study was conducted, increased isolation yields have been shown using an antibody against the mesenchymal marker N-cadherin (68), and using an enrichment strategy combining different antibodies specific for surface proteins and extracellular matrix (69). Unfortunately, no commercially available isolation method combining epithelial and mesenchymal markers was available at the time of the present study. An expanded panel of antibodies specific for epithelial as well as mesenchymal markers should be considered when designing future studies to potentially improve the yield of the proposed isolation protocol. As mentioned, FACS is a method that would allow simultaneous isolation and sorting of different populations of CTCs.

An additional limitation of the MACS technology is its feasibility in clinical practice, mostly due to the duration of the isolation process, as it has been previously noted (70, 71). Together with time required for cytospin preparation and staining, the total processing time can extend to several hours. Moreover, constant supervision is required since most of the procedures are not automated. The implementation of the proposed method into clinical practice would, despite existing infrastructure and the ease of use of the technology itself, require organizational changes, namely in terms of requiring more personnel. Considering CTC analysis has not yet been incorporated into clinical practice guidelines, and its utility despite many other potential applications remains mostly prognostic, the proposed isolation technology does not appear to be sufficiently cost-effective.

## Conclusion

The non-invasiveness of the liquid biopsy compared to other diagnostic methods for tumor tissue analysis has sparked great interest for its implementation in clinical practice. The main limitations in the development of isolation technologies are lengthy processing times and low sensitivity. However, in the clinical setting, preservation of morphology by the isolation procedure is of particular interest, as it may enable cytopathological evaluation with existing resources. Due to the morphological degeneration of isolated breast cancer CTCs, which could be due either to apoptotic cell death, the isolation process, or to sheer forces in the bloodstream, we believe the MACS technology followed by preparation of standard cytological slides is not suitable for use in a clinical cytopathology laboratory for isolation of CTCs from the blood of early or locally advanced breast cancer patients. Nevertheless, considering the high isolation specificity and the fact that the MCF7 cells were well preserved following the isolation protocol, the method could well be used for research purposes for enrichment of specific cell types or in studies where cytological samples are studied.

## Data Availability Statement

The raw data supporting the conclusions of this article will be made available by the authors, without undue reservation.

## Ethics Statement

The studies involving human participants were reviewed and approved by the National Medical Ethics Committee at the Slovenian Ministry of Health. The patients/participants provided their written informed consent to participate in this study.

## Author Contributions

CGK, TJ, VKP, and MC: conceptualization. CGK and TJ: methodology. TL, TJ, VH, AJ, NN, and VKP: analysis and investigation. CGK: resources. TL: writing – original draft preparation. TJ, CGK, MC, VKP, and NN: writing – review and editing. All authors have read and agreed to the published version of the manuscript.

## Conflict of Interest

The authors declare that the research was conducted in the absence of any commercial or financial relationships that could be construed as a potential conflict of interest.
